# Borderline tuberculoid leprosy mimicking sarcoidosis: A case report

**DOI:** 10.1097/MD.0000000000011616

**Published:** 2018-08-10

**Authors:** Jian Liu, Yan Wen, Yan Xing, Shuo Wang

**Affiliations:** a Beijing Tropical Medicine Research Institute, Beijing Friendship Hospital; b Beijing Key Laboratory for Research on Prevention and Treatment of Tropical Diseases (BZ0086), Capital Medical University, Beijing, China; c Pacific Institute of Medical Sciences, Bothell, Washington.

**Keywords:** diagnosis, granulomatous disease, leprosy, sarcoidosis

## Abstract

**Introduction:**

Leprosy is a chronic infectious granulomas disease caused by *Mycobacterium leprae* that can manifest as a wide variety of immunological and clinical features.

**Case summary:**

Here, we describe the case of a woman with clinical characteristics of borderline tuberculoid (BT) leprosy that manifested as 3 asymmetric skin lesions involving her hip and lower limbs. This unusual presentation was initially misdiagnosed as sarcoidosis because noncaseating granulomas are a histopathological feature of both diseases. Differentiation and the diagnosis of BT leprosy was achieved using real-time polymerase chain reaction (PCR) to amplify an *M leprae* specific DNA sequence and to detect serum antibodies specific to *M leprae* antigens. Accordingly, a 6-month course of multidrug therapy led to a marked improvement in the skin lesions.

**Conclusion:**

The use of auxiliary tests including real-time PCR to amplify an *M leprae*-specific DNA sequence, enzyme-linked immunosorbent assay, and dipstick detection of serum antibodies specific to *M leprae* antigens are good methods to obtain a correct diagnosis of BT leprosy.

## 1 Introduction

Leprosy or Hansen disease is a chronic granulomatous disease caused by the intracellular bacterium *Mycobacterium leprae* that invades skin and peripheral nerves.^[1]^ The disease is distributed throughout the world, mainly in Brazil, India, Southeast Asia, and Africa. Leprosy can manifest as a wide spectrum of clinical features, but the predominant manifestations are skin lesions with diminished sensation, thickened subcutaneous nerves, and the presence of acid fast bacilli. The Ridley-Jopling classification criteria,^[2]^ histopathologic findings, skin-slit smear (SSS) with acid-fast staining, and bacteria index (BI) categorize the disease into 5 types (tuberculoid leprosy [TT], borderline tuberculoid [BT], mid-borderline [BB], borderline lepromatous [BL], and lepromatous leprosy [LL]). In polar TT cases, skin patch lesions manifest as slightly hypochromic, round macules with discontinuously populate, well-bordered, lesions insensitive to touch, and a low BI. The extreme LL form is characterized by a symmetrical distribution, diffuse infiltration, nodules, and madarosis, with a higher number of infiltrated skin lesions and high BI compared with the other types. Borderline leprosy is characterized by the appearance of multiple irregular, thick granular margins and small satellite, coalescent lesions, with usually positive bacilloscopy results. Patients with paucibacillary (PB) leprosy (TT and BT) are treated for 6 months with a regimen consisting of rifampicin and dapsone. Given their increased bacteria load, patients with multibacillary (MB) leprosy (BB, BL, and LL) receive treatment for 12 months with clofazimine in addition to rifampicin and dapsone.

Based on the *M leprae*-specific cell-mediated immunity of the host, the types of leprosy from strongest to weakest responses are as follows: TT, BT, BB, BL, and LL.^[3]^ To guide treatment decisions, the World Health Organization (WHO) classified leprosy into the operational classifications of PB and MB leprosy according to the number of skin lesions and involved nerves.^[3]^ In terms of histopathologic findings, PB (TT and BT) is characterized by epithelioid granuloma cells, whereas MB (BB, BL, and LL) is characterized by maculated granuloma cells.^[1]^ Leprosy is currently diagnosed by a physical examination, and when possible, a microbiological evaluation using SSS.^[4,5]^

Here, we describe a case of BT leprosy that was originally mistaken for sarcoidosis. We discuss the clinical and histologic characteristics of the disease and the methods used to obtain the correct diagnosis.

## 2 Case report

Informed consent was obtained from the individual participant in the study.

**Figure 1:**
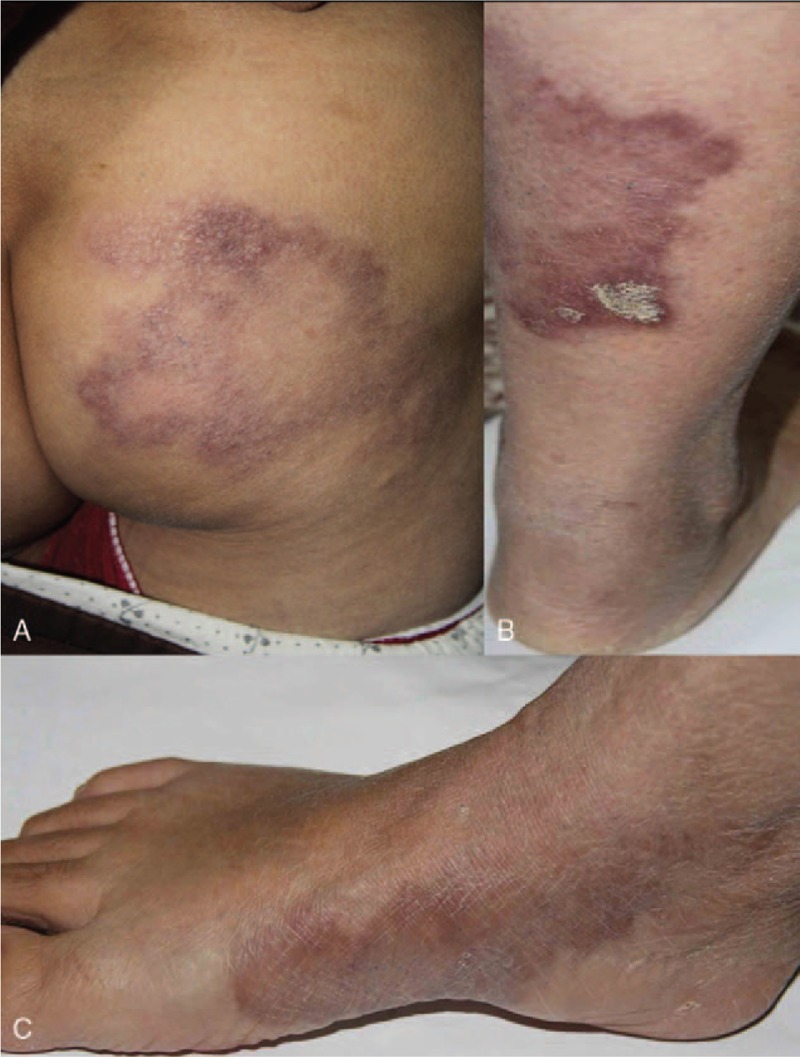
Macroscopic presentation of the plaques. (A) Palm-sized plaques are shown on the right hip. These plaques lacked a clear edge, and the fringes were raised higher than the skin. Normal skin is occasionally observed among the patches. (B) On the right leg, palm-sized plaques are present with varying shades of color, deep and shallow edges, and satellite foci. (C) On the inside of the right foot, the plaque has dark coloring, undefined edges, and satellite foci.

**Figure 2:**
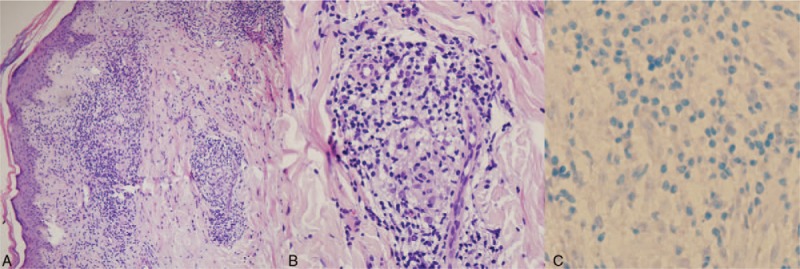
Microscopic observations of the skin biopsies. (A) Under 100× magnification, thinning of the skin is not clearly seen, infiltration is not obvious, and shallow blood vessels are visible around the granulomatous adnexal tissue in the mid-dermis. (b) Under 400× magnification, granulomatous changes are visible, with epithelioid cells and lymphocyte infiltration. (c) Under 400× magnification, sections show a negative result with acid-fast staining.

A 40-year-old woman who worked as a peasant farmer in Shandong province, 300 miles from Beijing, China, was referred to a dermatology clinic within that province because she had 3 patchy rashes (Fig. 1A–C). One year earlier, she had 3 large patchy rashes—1 on the hip and 2 on her lower limbs. The patch on the hip was wide, raised, and erythematous with well-defined margins sloping toward the center of the lesion. The erythematous patch on the dorsum of her foot had sharp edges with thick granular margins and small satellite lesions. A skin biopsy was performed and revealed a noncaseating epitheloid granuloma (Fig. 2A). On the basis of this observation, we diagnosed the patient as having cutaneous sarcoidosis. She was prescribed some herbal medicine for 1 month, after which she felt aggravated and visited the hospital again. Then a skin biopsy from the patch on her right leg was obtained. Similar to the previous biopsy, the result showed numerous noncaseating epithelioid granuloma cells consisting of histiocytes and giant cells surrounded by lymphocytes infiltrating the adnexa structures (Fig. 2B, C). Results of the Ziehl-Neelsen staining and polymerase chain reaction (PCR) test for *Mycobacterium tuberculosis* were negative, and routine laboratory screening test results for the hemoglobin level, leukocyte count, and liver and renal parameters were all within normal range. Because of the possibility of sarcoidosis, plasma dipeptidyltransferase and calcium levels were measured, and chest radiography was performed. All these test results were also normal. Consequently, leprosy was suspected, and the patient was referred to Beijing Tropical Medicine Research Institute (BTMRI) for a formal assessment.

By the time of referral to BTMRI, the 3 patches became asymmetrically distributed on the patient's hip and lower limbs, while no patches were founded on her trunk or scalp. All the patches were not sensitive to light touch, and the patient was unable to discriminate between hot and cold temperatures in these areas. The eye examination was normal, and there was no enlargement of the nervus auricularis magnus. Subsequently, SSS with acid-fast staining was performed at 5 different sites: both earlobes, both eyebrows, and the chin. Results of the microscopic evaluation were negative. Therefore, *M leprae*-specific real-time PCR and enzyme-linked immunosorbent assay were also performed to detect serum antibodies against phenolic glycolipid (PGL)-I and leprosy IDRI diagnostic (LID)-1. The results of real-time PCR for an *M leprae*-specific target was positive, whereas the antibody responses against LID-1 and PGL-I mimetic NDO-BSA were positive but weak (OD >0.05) (Table 1). On the basis of the patient's clinical presentation and results of the assays, we diagnosed the patient as having BT leprosy. Accordingly, we prescribed her with dapsone (100 mg daily) and rifampicin (600 mg monthly) for 6 months in accordance with the WHO recommendation for treating PB leprosy.

**Table 1:**
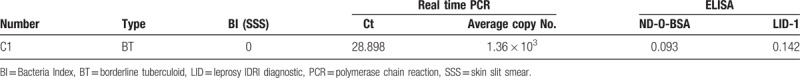
Results of the laboratory assays.

**Figure 3:**
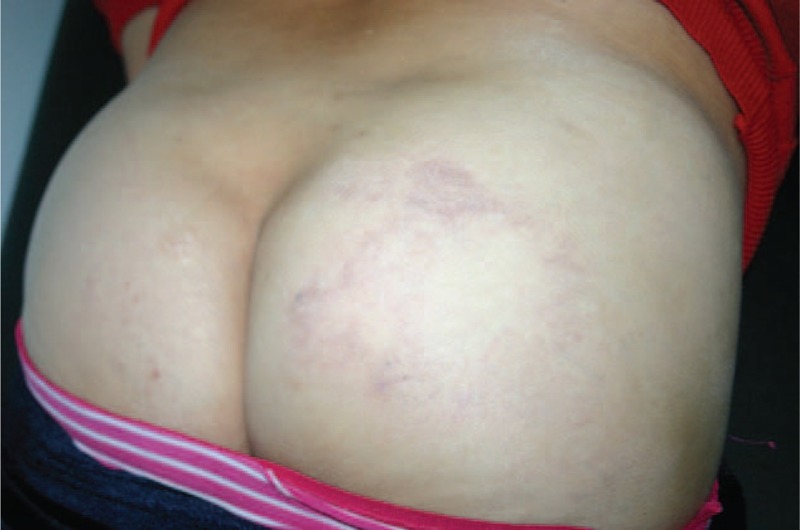
Recovery of the skin lesions following multidrug therapy (MDT). After a 6-month course of MDT for PB leprosy, the lesions show improvement to a more normal profile. PB = paucibacillary.

**Figure 4:**
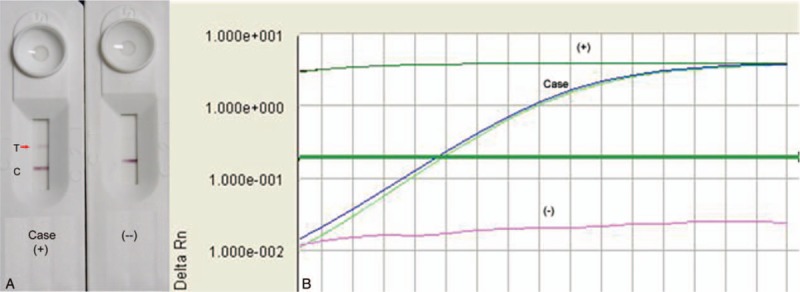
Samples from the case generate (A) a positive band in a rapid lateral flow test based on the detection of antibodies against the NDO-leprosy IDRI diagnostic conjugate antigen and (B) yield a *M leprae*-specific fragment amplified by real-time polymerase chain reaction.

The skin lesion was markedly improved by completion of the 6-month multidrug therapy (MDT) (Figs. 3 and 4A, B), providing a retrospective conformation of the diagnosis of BT leprosy.

## 3 Discussion

Sarcoidosis is a multisystem noncaseating granulomatous disease that affects organs, such as the lungs, skin, lymph nodes, eyes, and liver. The etiology of sarcoidosis is unknown at present, and although some cases are self-limiting, the prognosis is usually good with corticosteroids. Similar to leprosy, the most common histopathological feature of sarcoidosis is a kind of epithelioid cell granulomas. Some cases of PB leprosy may be confounded with sarcoidosis.^[6]^

Auxiliary tests are now becoming available to assist in the diagnosis of leprosy. Examination of the sera is useful for detecting antibodies against LID-1 and LID-NDO with high sensitivity and specificity to aid in the diagnosis of leprosy and help in identifying people with asymptomatic *M leprae* infection before lesion development. We found LID-1 to be an important parameter in the detection of new cases of leprosy, even in detecting a proportion of patients with PB leprosy.^[2,7]^ PCR-based tests can improve sensitivity of the PB diagnosis. Real-time PCR detection of *M leprae* in DNA could be superior to nested-PCR assay, which is prone to contamination with PCR products in the second round of amplification, and real-time PCR can be used as a rapid, sensitive, and specific confirmatory test to identify the presence of *M leprae* in tissue specimens for diagnosing PB leprosy.^[8–10]^

The patient with BT leprosy described herein seldom had the clinical characteristics of sarcoidosis, such as hypercalcemia, a high globulin level, hematic disease, a low lymphocyte count, leukemia, rapidly increased blood sedimentation level, circulating immune complex deposition, but she did have some clinical symptoms of sarcoidosis, including skin numbness, pain/warm touch sensory disorder, and numb areas near the skin lesion. Therefore, she was diagnosed as having sarcoidosis at her local clinic in the Shandong province. After a period of ineffective treatment and flaring of the lesions, the patient was referred to BTMRI under the suspicion of leprosy. The clinical examination was conducted by our experienced clinicians and additional diagnostic assays were performed. Although the microbiology of SSS and histopathologic results were negative, antibodies against both LID-1 and PGL-I (NDO-BSA) were detected at low levels, and real-time PCR amplifying a *M leprae* gene fragment was positive. A diagnosis of BT leprosy was made, and after providing MDT for 6 months, the skin lesions showed marked improvement.

## Acknowledgment

The authors express gratitude to Senior Scientist Malcolm Duthie, Ph.D. for editing and revising this manuscript.

## Author contributions

**Formal analysis:** Yan Wen.

**Project administration:** Jian Liu.

**Supervision:** Yan Xing.

**Writing – original draft:** Jian Liu.

**Writing – review and editing:** Shuo Wang.
